# *Mycobacterium marinum* Hand Infection in a “Sushi Chef”

**Published:** 2009-10-14

**Authors:** David J. Cennimo, Richard Agag, Earl Fleegler, Alfred Lardizabal, Kenneth M. Klein, Cornelia Wenokor, Shobha Swaminathan

**Affiliations:** ^a^Division of Infectious Diseases, Department of Medicine, New Jersey Medical School, University of Medicine and Dentistry of New Jersey; ^b^ Division of Plastic Surgery, Department of Surgery, New Jersey Medical School, University of Medicine and Dentistry of New Jersey; ^c^ Division of Pulmonary and Critical Care Medicine, Department of Medicine, New Jersey Medical School, University of Medicine and Dentistry of New Jersey; ^d^ Department of Pathology and Laboratory Medicine, New Jersey Medical School, University of Medicine and Dentistry of New Jersey; ^e^ Department of Radiology, New Jersey Medical School, University of Medicine and Dentistry of New Jersey

## Abstract

**Objective:** We present the case of a sushi chef with pain and swelling of his index finger and wrist for a year, unresponsive to antibiotics. **Methods:** Biopsy showed a xanthogranulomatous reaction and positive culture results for *Mycobacterium marinum*. **Results:** He was treated with minocycline, clarithromycin, and ethambutol. In addition, he underwent radical synovectomy of the lesion. **Conclusion:** The combined medical and surgical approach resulted in a positive outcome.

## CASE PRESENTATION

A 41-year-old man presented with a painful, swollen right index finger with decreased range of motion and pain in the ipsilateral wrist. He denied trauma but reported clear fluid draining intermittently from the finger. He had taken 2 courses of cephalexin and 1 course of oral penicillin with no relief. Two incision and drainage procedures were previously attempted without improvement.

The patient, born in China, immigrated to the United States 10 years ago. He was working as a sushi chef and admitted to multiple cuts and abrasions to his left hand but none to the right. His significant medical history included treatment of tuberculosis at 5 years of age. Surgical history included the incision and drainage with negative culture results. He was taking no medications and denied any allergies, smoking, alcohol, or illicit drug use. A review of systems was otherwise unremarkable.

Upon examination, he was afebrile and healthy appearing. There was minimal erythema but marked edema around the right index finger without fluctuance or drainage. A significant decrease in the range of flexion/extension at the metacarpophalangeal joint, proximal interphalangeal joint, and distal interphalangeal joint of the right index finger was noted. There was intact abduction and adduction of the digits and no pain on passive extension. A nontender, fluctuant swelling involving the mid-volar aspect of the wrist was present. No lymphadenopathy was appreciated and sensation was intact. The remainder of his physical examination was normal.

**Figure 1:**
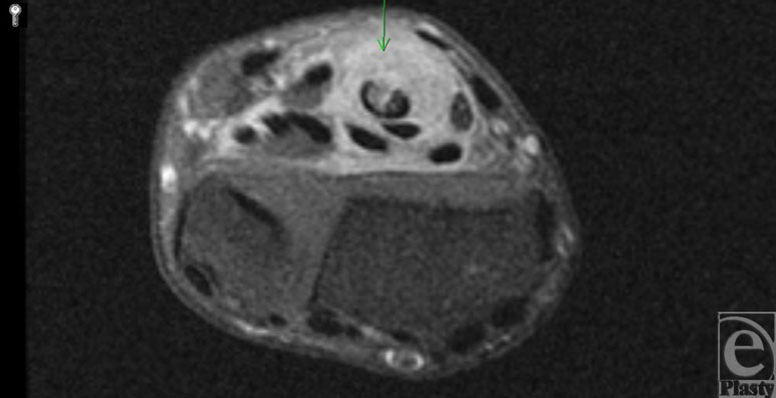
Axial post contrast fat-suppressed T1-weighted MRI through the distal forearm demonstrates extensive synovial enhancement of the flexor tendons with a partial tear of the flexor digitorum superficialis tendon of the middle finger (arrow).

**Figure 2:**
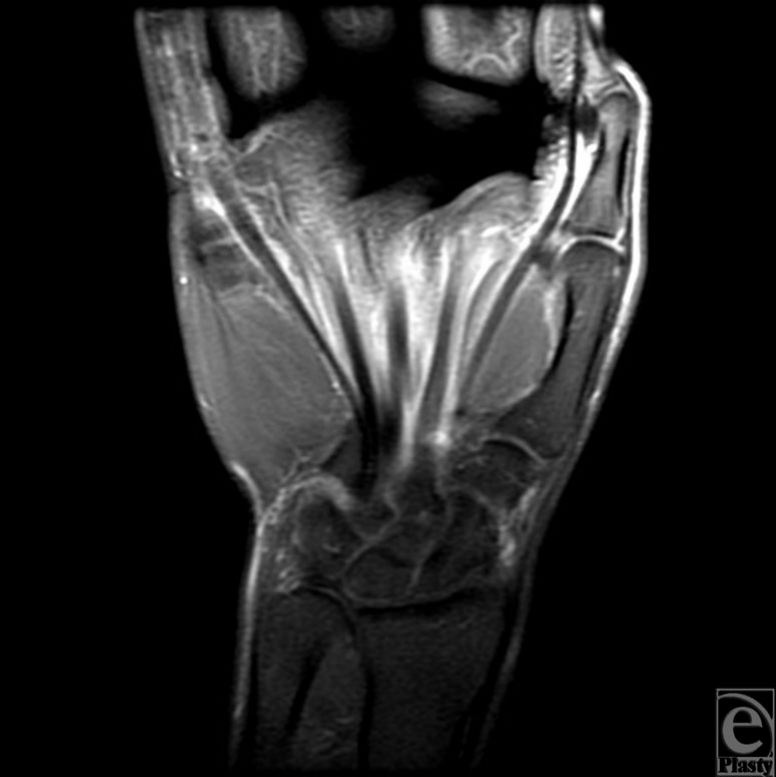
Coronal post contrast fat-suppressed T1-weighted MRI demonstrates communication between the common flexor tendon sheath and the tendon sheath of the flexor pollicis longus tendon.

Radiography of the hand showed extensive soft tissue swelling of the index finger, more pronounced on the ulnar side. Magnetic resonance imaging revealed an ill-defined abscess collection and phlegmon involving the index finger tracking into the wrist with small fluid collections deep to the flexor tendons and stretching of the A2 pulley (Figs 1 and 2). There was also marked tenosynovitis of the index, middle, and ring finger flexor tendons.

**Figure 3:**
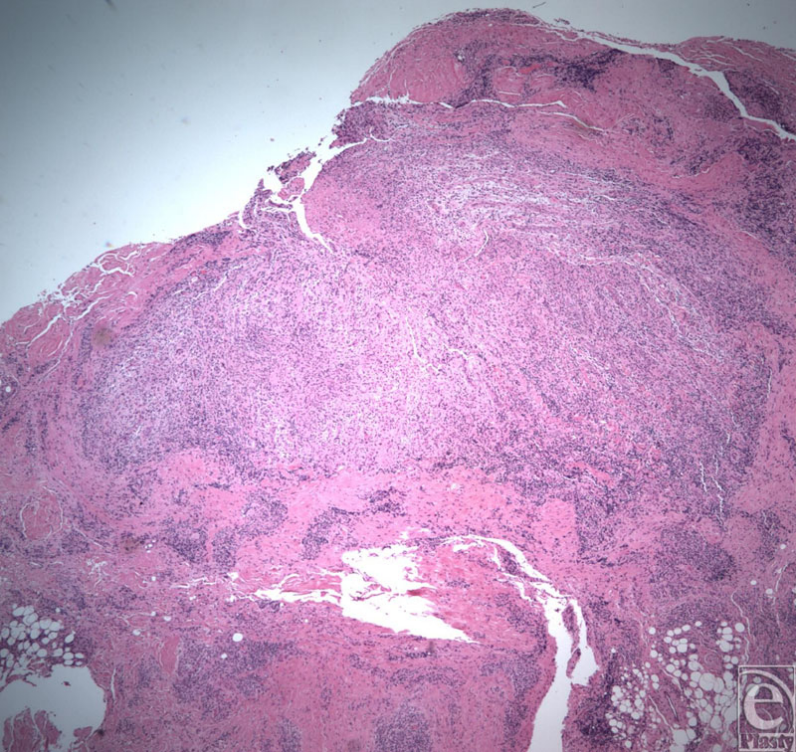
Non caseating granuloma (lower power).

**Figure 4:**
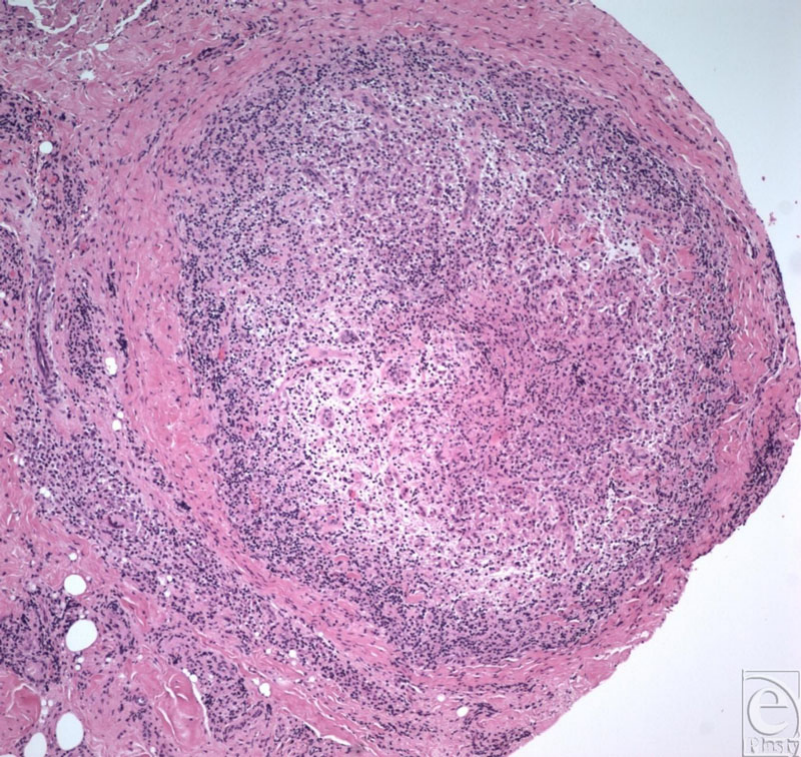
Non caseating granuloma (higher power).

All the laboratory examinations including a complete blood cell count, chemistries, and sedimentation rate were normal. Tuberculin skin testing gave positive results at 20 mm of induration. The patient underwent a biopsy that showed fibroconnective tissue with nonspecific chronic inflammation (Figs 3 and 4). No caseating granulomas were seen. Special stains were negative for acid-fast bacteria and fungi. A tissue polymerase chain reaction was negative for *Mycobacterium tuberculosis*.

Given the clinical presentation and the patient's occupation, *Mycobacterium marinum* was presumed the most likely pathogen. However, his history of tuberculosis with uncertain treatment prompted initial broad-spectrum treatment of mycobacteria until culture results were available. He began isoniazid, rifampin, pyrazinamide, ethambutol, and clarithromycin. Cultures incubated at room temperature on Lowenstein-Jensen medium grew *M. marinum* after 5 weeks. Therapy was changed to minocycline, clarithromycin, and ethambutol. The patient refused to continue rifampin because of the urine discoloration and minocycline was substituted. A 3-drug regimen was felt to represent maximal antibiotic therapy with the least possibility for resistance and favored because of the depth of infection. The patient subsequently underwent a tenosynovectomy. Intraoperative findings included a creamy yellowish, chronically inflamed-appearing tissue adherent to the underlying median nerve. Fraying of an involved flexor tendon was also noted. The adjacent anatomic features were obscured by a diffuse, chronic inflammatory-appearing tissue, and an “oily” tan-colored fluid was encountered during dissection.

A repeat magnetic resonance imaging 6 months later showed complete resolution of the previously seen abscess and tenosynovitis. Six months after completing antibiotics, he developed a painful swelling on his right index finger. A soft, mildly tender nodule about 1 cm in diameter and minimal swelling at the ipsilateral wrist were appreciated. Given the concern for recurrent mycobacterial infection, the patient underwent repeat exploration of the area. A “caseous” material was found within the mass excised from the right index finger and the distal phalanx extensor tendon area. Excision of the mass and associated chronic synovitis was carried out from the right carpal tunnel/distal forearm areas. Pathology revealed an epidermoid inclusion cyst of the finger, a nodular histiocytic reaction (similar to a xanthogranuloma), and foreign body granuloma of the right wrist. The patient was resumed antimycobacterial medications pending culture results. All cultures were negative for mycobacteria; however, given the surgical findings indicating a high suspicion for infection, antibiotics were continued for 6 months. The patient experienced improvement in the swelling and range of motion of his finger and wrist. He received a total of 12 months of antibiotic therapy.

## DISCUSSION

*M. marinum* was initially isolated in 1926 as a fish pathogen in a Philadelphia aquarium. Human infection was first described in a swimming pool outbreak in 1954 and has led to the terms “fish tank granuloma” and “swimming pool granuloma.”^1^–^3^ *M. marinum* is found worldwide in both fresh and salt water. It is a phytochromogen and grows optimally at 30°C to 32°C. Human infections have been associated with fish, shellfish, and swimming.^2^,^3^ We present a case of likely occupational exposure in a sushi chef in whom the diagnosis was missed for over a year.

The estimated annual incidence of infection is between 0.05 and 0.27 cases per 100,000.^1^,^3^,^4^ A review of 66 infections from France documented exposure to fish tanks to 84%, with a majority reporting deaths in the fish populations.^4^ Jernigan and Farr^5^ reported exposures in 193 cases as follows: 49.2% aquarium; 27.4% fish or shellfish preparation; 8.8% injury in water; and 2.6% swimming pools. Although skin breaks are necessary for infection, they can be minimal. Most exposures do not result in disease.^3^ Unlike other mycobacterial infections, immunocompromise has not been identified as a significant risk factor in *M. marinum* infection.^1^,^6^

The clinical presentation varies, but infections are usually confined to the extremities because of the organisms' temperature requirements for optimal growth. Infections have been classified into 3 types. Type I lesions are small (1–2 cm), violaceous papules representing superficial infection. Type II lesions are subcutaneous granulomas, which may be multiple and can ulcerate. Type III lesions (as was the case in our patient) represent deeper infections such as abscess, tenosynovitis, septic arthritis, bursitis, or osteomyelitis^1^–^3^ and have been reported in up to 30% of cases.^7^ A phenomenon of painless sporotrichoid spread has been described in 20% to 75% of cases.^2^,^3^,^8^ The incubation period is approximately 3 weeks. Jernigan and Farr^5^ confirmed a mean incubation period of 21 days, with a range of 5 to 270 days; 90% had an incubation period of less than 60 days.

The diagnosis can be difficult and begins with a thorough history for exposure. Because of the prolonged incubation, probe for water and fish/shellfish exposure up to 9 months before clinical presentation. There are no pathognomonic aspects of the presentation, but most case series report no fever, leukocytosis, lymphadenopathy, or elevated inflammatory markers.^1^,^8^ The diagnosis is usually made after biopsy and culture of the lesion, but the microbiologist should be alerted to the possibility of *M. marinum*.^2^ Cultures should be done at 30°C for optimal results. Infected synovium or “rice bodies” can be cultured because they contain live mycobacteria.^9^ Microbiologic stains give positive results in less than 25% of cases and do not correlate with the severity of infection.^3^ Similarly, granulomas are seen in less than 50% of biopsy specimens and rarely in the first 6 months of infection.^2^,^3^

*M. marinum* can give positive results for tuberculin skin testing.^8^ However, because of his positive results for tuberculin skin testing (either from prior tuberculosis or from *M. marinum* infection), our patient initially started treatment of both mycobacteria until culture reports were finalized.

The optimal treatment has yet to be defined. *M. marinum* is intrinsically resistant to pyrazinamide and many isolates are also resistant to isoniazid.^2^ Most case reports present good outcomes with a prolonged course of multiple antibiotics. The most common treatment is a combination of ethambutol and rifampicin. Good results have been shown with clarithromycin, tetracyclines, trimethoprim/sulfamethoxazole, and fluoroquinolones. Some data suggest higher risk of treatment failure if ciprofloxacin is used and instead favor moxifloxacin, which has better activity.^10^ Currently, combinations of clarithromycin and a second agent (such as a tetracycline) are gaining popularity. The course of therapy is generally extended for 1 to 2 months after resolution of symptoms and has ranged between 2 months and 2 years.^1^,^2^,^4^,^8^ The current American Thoracic Society/Infectious Diseases Society of America guidelines for the treatment of nontuberculous mycobacteria do not recommend routine antibiotic susceptibility testing outside of clinical treatment failure.^10^

Our patient was diagnosed with tenosynovitis of the hand, carpal tunnel, and forearm. Because of the deep-seated nature of this chronic infection and tendons involvement, there was concern that the poorly vascularized, fibrotic tissue would prevent adequate antibiotic perfusion in the infected area. Therefore, a tenosynovectomy, although controversial, was performed.^9^ The opinion on synovectomy for atypical mycobacterial infection of the upper extremity varies in the literature from “radical synovectomy may not be necessary”^11^ to “extensive surgical debridement is recommended.”^12^ Our decision to perform a radical synovectomy was based on the extent of the bulky, poorly vascularized, fibrotic, inflammatory tissue and the estimation of adequate antibiotic penetration.

## CONCLUSION

Despite awareness of atypical mycobacterial infections, diagnosis is frequently delayed leading to increased morbidity. Patients with an exposure to these atypical pathogens, in this case being a sushi chef, require a broadened differential to include appropriate testing and appropriate culture of specimens to obtain an accurate diagnosis.
